# Association between Serum Zinc and Calcification Propensity (T_50_) in Patients with Type 2 Diabetes Mellitus and In Vitro Effect of Exogenous Zinc on T_50_

**DOI:** 10.3390/biomedicines8090337

**Published:** 2020-09-09

**Authors:** Shinya Nakatani, Katsuhito Mori, Mika Sonoda, Kozo Nishide, Hideki Uedono, Akihiro Tsuda, Masanori Emoto, Tetsuo Shoji

**Affiliations:** 1 Department of Metabolism, Endocrinology and Molecular Medicine, Osaka City University Graduate School of Medicine, 1-4-3 Asahi-machi, Abeno-ku, Osaka 545-8585, Japan; m2026719@med.osaka-cu.ac.jp (S.N.); mksnd1110@gmail.com (M.S.); westoutdedede@yahoo.co.jp (K.N.); uedono1217@yahoo.co.jp (H.U.); naranotsudadesu@infoseek.jp (A.T.); memoto@med.osaka-cu.ac.jp (M.E.); 2 Department of Nephrology, Osaka City University Graduate School of Medicine, 1-4-3 Asahi-machi, Abeno-ku, Osaka 545-8585, Japan; 3 Division of Internal Medicine, Inoue Hospital, 16-17 enoki-machi, Osaka 564-0053, Japan; 4 Department of Vascular Medicine, Osaka City University Graduate School of Medicine, 1-4-3 Asahi-machi, Osaka 545-8585, Japan; t-shoji@med.osaka-cu.ac.jp; 5 Vascular Science Center for Translational Research, Osaka City University Graduate School of Medicine, 1-4-3 Asahi-machi, Abeno-ku, Osaka 545-8585, Japan

**Keywords:** serum calcification propensity, zinc, type 2 diabetes mellitus

## Abstract

Zinc inhibits vascular calcification in vivo and in vitro. Patients with type 2 diabetes mellitus show hypozincemia and are at an elevated risk of cardiovascular events. Recently, an in vitro test (T_50_-test) was developed for determination of serum calcification propensity and a shorter T_50_ means a higher calcification propensity. This cross-sectional study investigated the association between serum zinc and T_50_ in 132 type 2 diabetes mellitus patients with various kidney functions. Furthermore, the effect of exogenous zinc on T_50_ was also investigated in vitro using separately pooled serum samples obtained from healthy volunteers and patients with hemodialysis. We measured T_50_ levels using the established nephelometric method. The median (interquartile range) levels of T_50_ and serum zinc were 306 (269 to 332) min, and 80.0 (70.1 to 89.8) µg/dL, respectively. Serum zinc level showed a weak, but positive correlation with T_50_ (*r_s_* = 0.219, *p* = 0.012). This association remained significant in multivariable-adjusted analysis, and was independent of known factors including phosphate, calcium, and magnesium. Kidney function and glycemic control were not significantly associated with T_50_. Finally, in vitro experiments showed that addition of a physiological concentration of exogenous zinc chloride significantly increased serum T_50_. Our results indicate that serum zinc is an independent factor with a potential role in suppressing calcification propensity in serum.

## 1. Introduction

Vascular calcification is common and contributes to cardiovascular mortality in patients with type 2 diabetes mellitus [1,2], as well as those with chronic kidney disease [3,4]. Excess cardiovascular morbidity and mortality in those patients could be explained by redistribution and/or overload of calcium and phosphorus. Primarily, the mechanism of vascular calcification is supposed to be due to ectopic deposition of hydroxyapatite [5,6] induced by increased calcium-phosphorus product (Ca × P) in serum [7,8], which are common under renal dysfunction. In addition, imbalanced-calcification regulators may be involved in vascular calcification [3,9]. For example, bone morphogenetic protein-2 (BMP-2), oxidized lipids, and inflammation are known to accelerate vascular calcification [10]. In contrast, it has been reported that matrix Gla protein (MGP), osteoprotegerin, and osteopontin act on the vascular wall as calcification inhibitors [11].

Intense research has also suggested active regulated mechanisms of vascular calcification similar to osteogenesis. Previous studies reported trans-differentiation of vascular smooth muscle cells (VSMCs) into osteoblast-like cells [12,13]. During the process of vascular calcification, VSMCs seem to lose their original contractile phenotypes and alternatively express bone-related genes. Indeed, expressions of BMP-2 and osteopontin were confirmed in human arterial walls. In contrast to these extracellular factors, recent work focused on runt-related transcription factor 2 (Runx2), an essential transcriptional factor in osteogenesis. Runx2 appears to be involved in repression of the primary VSMC phenotype, in addition to acceleration of the osteogenic phenotype [14].

Except for trans-differentiation of VSMCs, degradation of matrix proteins such as elastin, may be associated with the progression of vascular calcification. That is, degradation of elastin by metalloproteinases (MMPs) provides a scaffold for precipitation of hydroxyapatites. Notably, high-glucose condition might accelerate vascular calcification through elastin degradation by MMPs. Compared to subjects without diabetes, patients with diabetes have upregulated MMPs in the arterial wall and higher plasma levels of MMPs [15,16]. Therefore, it is speculated that diabetic condition and renal dysfunction share some common causal pathways leading to vascular calcification.

In serum, precipitation of supersaturated calcium and phosphate is prevented by the formation of amorphous primary calciprotein particles [17,18]. Primary calciprotein particles spontaneously convert into secondary calciprotein particles, containing crystalline hydroxyapatite [17,18]. An in vitro test (T_50_-test) for determination of serum calcification propensity has been developed [19]. This assay measures time required for primary calciprotein particles to transform into secondary calciprotein particles in the presence of supersaturating doses of calcium and phosphate, which increase turbidity of samples. Serum T_50_ can be measured by laser light scatter in turbid samples using nephelometry. Thus, a shorter T_50_ means a higher calcification propensity. Previous studies have shown that lower T_50_ predicts vascular stiffness progression and all-cause mortality in patients with chronic kidney disease stage 3 and 4 [20], and all- cause mortality and cardiovascular composite endpoint in hemodialysis patients [21]. A lower T_50_ was also shown to predict cardiovascular and all-cause mortality in renal transplant recipients [22,23]. While it remains unclear how well in vitro T_50_ assay results represent the mineralization process in vivo. T_50_ results have been shown to represent arterial calcification, arterial stiffness, cardiovascular outcomes, and mortality in at least 18 observational and 11 interventional studies [24]. Thus, this assay is considered able to provide novel and clinically important information.

Serum T_50_ is dependent on the complex interplay of pro-calcifying (i.e., calcium and phosphate) and anti-calcifying serum components (i.e., magnesium and albumin) [19]. Among them, a higher serum phosphate level was the factor most closely associated with lower T_50_ [21,25]. Phosphate has been reported to induce calcification of VSMCs in vitro [26]. Hyperphosphatemia is a risk factor for vascular calcification and cardiovascular mortality, not only in patients with chronic kidney disease [27], but also in the general population [28]. Thus, suppression of phosphate-induced vascular calcification is clinically important.

Zinc, an essential micronutrient that has catalytic, structural, and regulatory roles [29,30], is absorbed by enterocytes via the intestinal zinc transporter and reaches the bloodstream via basolateral membrane zinc transporter 1, where it binds to albumin in plasma, and can be eliminated by different routes, such as urine and sweat [31]. In insulin-dependent diabetes mellitus patients, urinary zinc levels are higher as compared to healthy individuals, while gastrointestinal absorption of zinc has also been shown to be decreased in those diabetic patients [32]. In other studies, the level of zinc in blood was found to be lower in patients with type 2 diabetes mellitus as compared to non-diabetic subjects [33,34,35]. Zinc deficiency is involved in development and progression of diabetes and zinc supplementation can improve the status of diabetes mellitus and its related complications [36]. A previous meta-analysis of zinc supplementation in patients with type 2 diabetes mellitus provided supportive evidence showing its hypoglycemic and lipid lowering effects [37]. In addition, zinc was recently found to inhibit phosphate-induced vascular calcification, in vitro and in vivo [38].

To date, no reports of association between serum zinc and calcification propensity in patients with type 2 diabetes mellitus have been presented. However, previous studies raise the hypothesis that zinc may be one of the factors affecting serum calcification propensity. To test the hypothesis, we examined the association between serum zinc and T_50_ levels in patients with type 2 diabetes mellitus. We also performed in vitro experiments to investigate the effect of increased zinc concentration on T_50_ in separately pooled serum samples obtained from healthy volunteers and hemodialysis patients.

## 2. Materials and Methods

### 2.1. Study Design

This study was comprised of two parts. The first was a cross-sectional study using clinical data derived from our previous study of 143 patients with type 2 diabetes mellitus [39], while the second part was an in vitro study of the effect of increased zinc concentration on T_50_ assay findings, which was conducted using separately pooled serum samples obtained from healthy volunteers and patients with hemodialysis. Such pooled serum samples are routinely measured as a quality control for the T_50_ assay. Since the in vitro experiments required a serum quantity of at least 1500 µL pooled serum samples were used.

### 2.2. Ethics Statement

This study followed the ethical guidelines for medical and health research involving human subjects by the Japanese Ministry of Health, Labour and Welfare, and the Declaration of Helsinki. This study was approved by the Ethics Committee of Osaka City University Graduate School of Medicine (approval number 4100, approved on 29 June 2018). Opt-out option for informed consent was performed as explained in instructions posted on the website of the institution.

### 2.3. Participants of the Cross Sectional Study

The inclusion and exclusion criteria for the clinical study were previously described [39]. Patients with type 1 diabetes or other types of diabetes were not included in this study. For this analysis, we excluded 11 participants because data regarding serum T_50_ and zinc were not available thus, 132 patients were included.

### 2.4. Physical and Laboratory Measurements, and Other Clinical Infromation

Blood pressure was determined using an automatic sphygmomanometer (Terumo Co., Ltd., Tokyo, Japan) with a conventional cuff after the subjects had rested for at least 5 min. All blood and urine samples were collected in the morning after an overnight fast for 12 h. Serum and urinary creatinine levels were measured by an enzymatic method. Urinary albumin was measured by an immunoturbidimetry. Serum zinc levels were measured by a commercial laboratory (SRL Co., Ltd., Tokyo, Japan). Hemoglobin A1c was assessed as the National Glycohemoglobin Standardization Program equivalent value (NGSP, %) according to the guidelines of the Japan Diabetes Society [40]. The diagnosis of type 2 diabetes mellitus was based on medical records and the criteria for diabetes mellitus as defined in the Report of the Expert Committee on the Diagnosis and Classification of Diabetes Mellitus [41]. Renal function was assessed by estimated glomerular filtration rate (eGFR) using a formula for the Japanese [42]. In this study serum calcium denotes calcium level adjusted for serum albumin according to Payne et al. [43]. Urinary albumin to creatinine ratio was calculated as an index of albuminuria. Other measurements were obtained using routine laboratory methods at Osaka City University Hospital.

We collected information on age, sex, height, weight, duration of diabetes, current medications, past history of cardiovascular disease (coronary artery disease, peripheral artery disease, aortic disease, and congestive heart failure requiring hospitalization), smoking habit, and laboratory data by asking the participants and/or by reviewing their medical records.

### 2.5. Devices, Plastic Materials, and Chemicals

The Nephelostar Plus microplate reader with 2 built-in Reagent injectors and MARS software was obtained from BMG Labtech (Offenburg, Germany) and 96-well plates and 96-well plastic covers were from Corning (Kennebunk, ME, USA). All chemicals (NaCl, Tris, Hepes, CaCl_2_, NaH_2_PO_4_, Na_2_HPO_4_, NaOH, ZnCl_2_) were pro-analysis -grade quality and purchased from Wako Pure Chemical Industries, Ltd. (Osaka, Japan).

### 2.6. Determination of Calcification Propensity (T_50_)

As previously reported [19], calcification propensity (T_50_) was evaluated by overloading of calcium and phosphate into serum, in vitro. According to the original method [19], we prepared three stock solutions as follows: (1) NaCl solution: 140 mM NaCl, (2) Calcium solution: 40 mM CaCl_2_ + 100 mM HEPES + 140 mM NaCl pH-adjusted with 10 M NaOH to 7.40 at 37 °C, and (3) Phosphate solute on: 19.44 mM Na_2_HPO_4_ + 4.56 mM NaH_2_PO_4_ + 100 mM HEPES + 140 mM NaCl pH-adjusted with 10 M NaOH to 7.40 at 37 °C. For preparation of 96-well plates, all solutions were prewarmed to 34.5 °C in a thermoconstant room.

Serum T_50_ levels were measured as follows: (1) All stock solutions were prewarmed to 34.5 °C, in a thermocnstant room, (2) In the 96-well plates, 20 µL of NaCl stock solution and 80 µL of serum were mixed in each well, (3) Shacked gently for 1 min, (4) Fifty µL of phosphate stock solution were added and shacked for 1 min, automatically, (5) Fifty µL of calcium stock solutions were added and shacked for 1 min. The final concentrations of calcium and phosphate in each sample were 10 mM and 6 mM, respectively, (6) The 96 wells were covers with a thin seal adhesive sealing film microplate. Because in the outermost lines and rows of the 96 well plate often showed unreliable result in the previous study, these lines were not used in the present study [19].

Serum T_50_ was determined in duplicate over a period of 600 min per measurement using a nephelometer (Nephelostar Plus^®^, BMG Labtech, Saitama, Japan) with an internal measurement temperature of 36.5 °C to 37 °C. The setting of the Nephelostar was described as previously [19]. The measurement results were analyzed by the MARS software (BMG Labtech, Saitama, Japan).

All serum samples were measured in a blinded manner. Serum samples from healthy volunteers and dialysis patients were also measured as quality control in serum calcification assay. The coefficients of variation (CV) of inter- and intra-assay were 4.4% and 4.5% for healthy control serum, and 3.2% and 4.5% for hemodialysis control serum.

### 2.7. In Vitro Experiment to Examine Direct Effect of Exogenous Zinc on T_50_

As the second part of this study, we conducted in vitro experiments to examine the direct effect of exogenous zinc on T_50_ measurement. For this, two pooled serum samples, one from healthy volunteers and another from hemodialysis patients, were used. As described above, 80 µL of each serum sample was mixed with 20 µL of NaCl stock solution, 50 µL of phosphate stock solution, and 50 µL of calcium stock solution in a 96-well plate. To the 200 µL of T_50_ assay mixture, 2 µL of one of the following solutions was added; (a) distilled water (vehicle, final zinc concentration = 0 µM), (b) 1 mM ZnCl_2_ (final zinc concentration = 10 µM), or (c) 2 mM ZnCl_2_ (final zinc concentration = 20 µM). After gentle agitation, the plate was sealed with thin film to prevent evaporation, and then incubation was performed at 36.5 °C to 37 °C for measurement of T_50_.

### 2.8. Statistics

For the clinical study, we summarized continuous variables as medians (interquartile ranges, IQRs) and categorical variables as numbers and percentages. Correlations were analyzed according to the nonparametric Spearman’s rank correlation test. Independent associations between the variables and serum T_50_ were assessed by multiple regression analysis. For in vitro experiments in which ZnCl_2_ was added, T_50_ was expressed as the mean (SD) of triplicate determinations, and comparison was made by one-way analysis of variance followed by Tukey’s test. These statistical analyses were performed using GraphPad Prism version 6.0 (GraphPad Software, San Diego, CA, USA) or JMP software version 10 (SAS Institute, Inc., Cary, NC, USA). *p*-values < 0.05 by two-sided tests were considered statistically significant.

## 3. Results

### 3.1. Clinical Characteristics of Type 2 Diabetes Patients

Table 1 summarizes the clinical characteristics of the 132 patients with type 2 diabetes mellitus. The age [median (interquartile range)] was 71 (65 to 75) years and 59.1% of the participants were men. Their eGFR [59.0 (37.6 to 73.9) mL/min/1.73 m^2^] and urinary albumin to creatinine ratio [17 (6 to 212) mg/gCr] showed wide distributions. T_50_ and serum zinc levels were 306 (269 to 332) min and 80.0 (70.1 to 89.8) µg/dL, respectively.

### 3.2. Correlations between Serum Calcification Propensity and Clinical Factors in Patients with Type 2 Diabetes Mellitus

**Figure 1:**
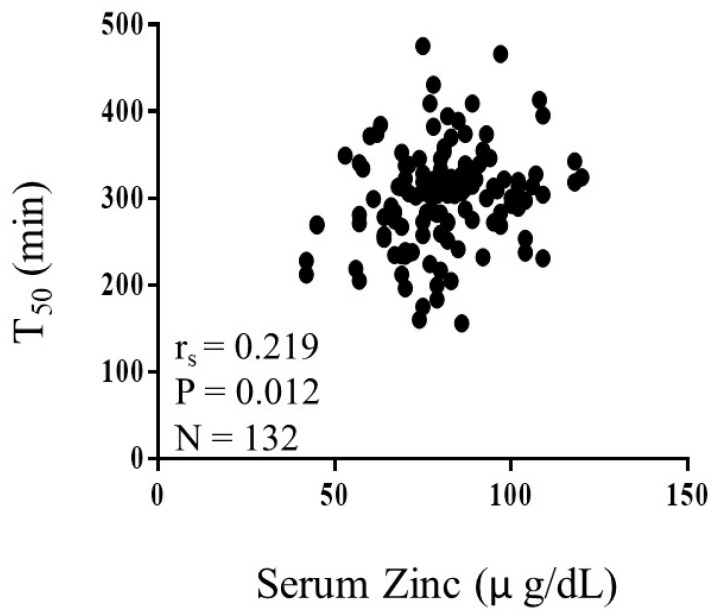
Correlation between serum zinc and serum calcification propensity (T_50_) in patients with type 2 diabetes mellitus. Serum zinc level showed a weak, but significant correlation with serum T_50_. Abbreviations: *r_s_*_,_ Spearman’s correlation coefficient; *p*, level of significance; N, number of patients.

Table 2 shows the unadjusted correlations between serum T_50_ levels and various clinical parameters in type 2 diabetes patients. While serum T_50_ was weakly, but positively correlated with zinc (*r_s_* = 0.219, *p* = 0.012, Figure 1), eGFR (*r_s_* = 0.199, *p* = 0.022), and fasting plasma glucose (*r_s_* = 0.282, *p* = 0.001), it was not significantly correlated with serum magnesium or hemoglobin A1c. Serum T_50_ level was negatively correlated with urinary albumin-creatinine ratio (r_s_ = −0.247, *p* = 0.004), blood urea nitrogen (*r_s_* = −0.213, *p* = 0.011), and serum phosphate (*r_s_* = −0.227, *p* = 0.009).

### 3.3. Independent Association between Serum Calcification Propensity and Zinc in Patients with Type 2 Diabetes Mellitus

We examined whether serum zinc level was a factor associated with serum T_50_, independent of traditional mineral makers including phosphate, calcium and magnesium, using multiple regression analysis (Table 3). Model 1 included age, sex, any prior cardiovascular disease, current smoking, urinary albumin creatinine ratio, eGFR, corrected calcium, phosphate, magnesium, zinc, and hemoglobin A1c as explanatory variables, with hemoglobin A1c being replaced by plasma glucose in Model 2. Urinary albumin, eGFR and hemoglobin A1c or fasting plasma glucose were not significantly associated with serum T_50_. In contrast, serum corrected calcium, phosphate, and magnesium were significantly associated with serum T_50._ Serum Zinc level was also found to be associated significantly and positively with T_50_ in both Model 1 (*β* = 0.213, *p* = 0.038) and Model 2 (*β* = 0.229, *p* = 0.024).

### 3.4. Influence of Zinc on Serum Calcification Propensity in Pooled Serum Samples Volunterres from Healthy Volunteers and Patients with Hemodialysis

**Figure 2:**
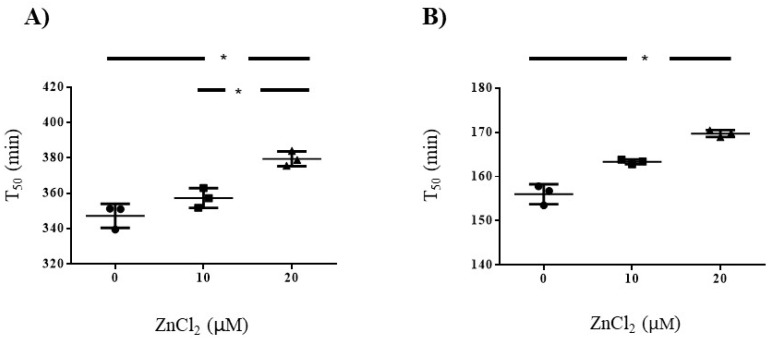
Influence of zinc on serum calcification propensity (T_50_) in pooled serum samples from healthy volunteers and patients with hemodialysis. Addition of exogenous 20 µM zinc chloride (ZnCl_2_) significantly increased T_50_. Compared to those of control (0 µM of ZnCl_2_ addition) in pooled serum from healthy subjects (**A**), and pooled serum from dialysis patients (**B**) * *p* < 0.05; statistically significant versus 0 µM ZnCl_2_ addition.

To examine whether zinc directly increases serum T_50_, zinc was added in the serum calcification propensity assay, in serum obtained from healthy volunteers and patients with hemodialysis, respectively. Supplemental Table S1 summarizes the clinical characteristics of the pooled serum samples from the two groups. Addition of exogenous ZnCl_2_ significantly modified the T_50_ level in pooled serum from healthy subjects (0 µM, 347 ± 0.8 min; 10 µM, 357 ± 5.6 min; and 20 µM, 379.5 ± 4.2 min; *p* < 0.001, Figure 2A), and pooled serum from dialysis patients (0 µM, 156 ± 2.3 min; 10 µM, 163 ± 0.5 min; and 20 µM, 170 ± 0.8 min, *p* < 0.001, Figure 2B), respectively.

## 4. Discussion

This is the first study to investigate the association between serum calcification propensity and zinc levels in patients with type 2 diabetes mellitus. Serum zinc level was significantly and positively correlated with serum T_50_ in the present study. The positive correlation between serum zinc level and T_50_ was also shown in the previous study including healthy subjects and patients with chronic kidney disease [38]. However, prior to the present investigation, whether serum zinc level is an independent factor associated with serum T_50_ was not examined. We showed that serum zinc level of the patients with type 2 diabetes mellitus was positively associated with T_50_ independent of calcium, phosphate, and magnesium, in the multiple regression analysis. These novel findings suggest that zinc has the potential role in suppressing calcification propensity in serum.

In the second part of the present study, we also confirmed that addition of zinc increases serum T_50_ in separately pooled serum obtained from healthy volunteers and patients with hemodialysis. However, the mechanisms underlying zinc inhibition of serum calcification propensity remain unclear. Even in polyethylene glycol hydrogels, not in serum, zinc was shown to inhibit the transformation of amorphous calcium phosphate (ACP) into hydroxyapatite [44]. In additive -free composite, ACP transformed into brushite within minutes. In contrast, in the presence of zinc, zinc-doped ACP was very stable and did not show any signs of crystallization for up to 20 days. In ACP, zinc ion readily substitutes calcium [45], suppressing crystallization by decreasing solubility [46]. It is thus likely that zinc suppresses the transformation from amorphous primary calciprotein particles into secondary calciprotein particles, containing crystalline hydroxyapatite, in serum.

In a recent study by Voelkl et al., addition of exogenous ZnCl_2_ (15 µM) did not improve T_50_ in sera from healthy controls or patients on hemodialysis [38]. The discrepancy between those result and the present may be explained by differences in ZnCl_2_ concentration. In the present study, we demonstrated that 10 µM ZnCl_2_ (=60.5 µg/dL) did not significantly modify T_50_ in serum from hemodialysis patients, which was consistent with the study by Voelkl et al. [38]. In contrast, ZnCl_2_ at 20 µM (=131 µg/dL), the upper limit of the reference range, significantly increased T_50_ in those subjects. Crystallization inhibition has been reported to be dependent on zinc concentration in polyethylene glycol hydrogels [44]. Thus, a certain zinc concentration may be required to increase serum T_50_, because addition of 10 µM of ZnCl_2_ in the present study or 15 µM of ZnCl_2_ in that previous study did not increase serum T_50_ [38].

In patients with type 2 diabetes mellitus, hypomagnesemia and hypozincemia are common, due to reduced intake and/or urinary loss [47,48,49]. In the present study, magnesium was also significantly and positively associated with serum T_50_. Magnesium is a known anti-calcifying factor, and has been shown to improve serum T_50_ in vitro [19], while other in vitro findings indicated that it can prevent phosphate-induced calcification in human aortic VSMCs [50]. Similarly, zinc increases zinc finger protein TNF-α-induced protein 3 (TNFAIP3) expression, which subsequently inhibits NF-kB activation and osteo-/chondrogenic reprograming, resulting in suppression of phosphate-induced VSMCs calcification [38]. Another report noted that zinc also inhibits osteochondrogenic phenotypic switch of VSMCs, reflected by reduced phosphate uptake, thus decreasing osteochondrogenic gene expressions of Msx-2, BMP-2, and Sp7, as well as loss of smooth muscle cell-specific markers [51]. That study also demonstrated that zinc preserves the phosphorylation state of Runx2 and Ser451, decreases the level of pyruvate dehydrogenase kinase 4 (PDK4) level, and restores cell viability (Supplemental Figure S1). Together, the present findings of the effect of zinc on serum T_50_, and the previous reported in vitro effects of zinc on phosphate-induced calcification in VSMCs [38,51], are similar to the effects of magnesium [50]. Although approximately 40% of magnesium binds to albumin or forms a complex with anions, including bicarbonate, phosphate, and citrate, in blood [52], a previous in vitro experiment demonstrated that addition of exogenous magnesium from 0.5 to 1.5 mmol/L increased serum T_50_ levels [19]. In addition, a recent randomized control trial demonstrated that magnesium supplementation increased serum T_50_ in patients with stage 3–4 chronic kidney disease [53]. Thus, supplementation with zinc as well as magnesium might be a potential therapeutic option to attenuate serum calcification propensity and the progression of vasculature calcification. Nevertheless, randomized clinical trials are clearly needed before such treatment is recommended.

Albumin is also an anti-calcifying factor associated with serum T_50_ in vitro [19]. When zinc and albumin were simultaneously included in multiple regression analysis, the significant associations of both factors with serum T_50_ turned to be non-significant (data not shown). Serum albumin acts as an extracellular zinc buffer that controls zinc concentration in blood, since approximately 75–80% of zinc is bound to albumin, accounting for as much as 98% of the exchangeable fraction of zinc in blood [54,55]. The present study showed that serum albumin is significantly and positively correlated with serum zinc levels in the study, thus a confounding effect might explain the results. Since albumin-bound zinc is exchangeable with other molecules, we speculate that serum albumin confounds the association between serum zinc and T_50_, at least in part, by an exchange between albumin-bound zinc and calcium.

Several studies have demonstrated that zinc supplementation of 30–100 mg per day increases serum zinc levels in patients with type 2 diabetes mellitus [56,57,58,59]. Meta-analyses of randomized controlled trials involving patients with type 2 diabetes mellitus revealed improvements in glycemic control [60] and dyslipidemia [61] following zinc supplementation. In patients with type 2 diabetes, the antioxidant effects of zinc supplementation have also been recognized [59]. In addition, a prior cohort study demonstrated that lower serum zinc level was an independent factor for coronary heart disease events in patients with type 2 diabetes mellitus [62]. Hence, the effects of zinc for glycemic control, lipid metabolism, antioxidants, and mineralization may contribute to suppress atherosclerosis and vascular calcification associated with type 2 diabetes mellitus. In contrast, other studies of general populations have shown that sustained hyperzincemia may predispose individuals to thrombogenesis [63], prostate cancer [64], and immune dysfunction [65]. In terms of atherosclerosis, there would be a trade-off between the thrombogenic adverse effect of zinc and its anti-arteriosclerotic effects including lipid metabolism and mineralization. Practical guidelines of zinc deficiency in Japan note the potential adverse effects of zinc therapy, including nausea, vomiting, itching, copper deficiency related anemia, and leukopenia [30]. Monitoring of serum zinc level is required to avoid these adverse effects including thrombosis in patients with zinc deficiency.

The present study has several limitations. First, the number of subjects examined and r values obtained in each correlation analysis were relatively low, possibly due to the limitation of the clinical study setting. Second, we cannot be sure whether the findings of this cross-sectional investigation are applicable to non-diabetic patients, or if the findings of in vitro experiments are also applicable for diabetic patients, since we did not perform in vitro experiments to add exogenous zinc to each patient in type 2 diabetes mellitus in the present study. Third, due to the cross-sectional design, the findings only demonstrate an association, not causality of the factor, i.e., zinc supplementation leads to increased serum T_50_ levels in patient with type 2 diabetes mellitus. To confirm the potential benefits of zinc supplementation, additional interventional studies are required. Fourth, we did not evaluate other serum markers that may influence serum T_50_. For example, iron has also been reported to be reduce high phosphate induced vascular calcification by inhibiting apoptosis [66]. Future research to investigate the association among serum iron, ferritin, transferrin saturation, and serum T_50_ is needed. Fifth, the method used for measurement of serum zinc level may be another limitation. In the present study, serum zinc levels were measured according to published practical guidelines for zinc deficiency in Japan [30]. However, fibrinogen contains several binding sites for zin ions [67,68]. Because some zinc might have been removed from the blood samples during extraction of the clots, measurement of zinc in plasma obtained with tubes containing a non-chelation-based anticoagulant might be better than the method used in our study [69]. Sixth, we did not measure plasma free fatty acid levels, though those are known to be elevated in patients with type 2 diabetes mellitus [70,71]. Albumin also binds to fatty acids [72]. Because the binding of zinc and fatty acids to serum albumin is linked allosterically [73], free fatty acids may have effects on modulation of plasma zinc speciation via an allosteric switch to serum albumin [74]. Finally, since pulse wave velocity (PWV) and intima-media thickness (IMT) were not evaluated, the relationship of calcium, magnesium, and zinc with atherothrombotic stage was not examined. While serum zinc was positively correlated with serum calcium and eGFR (*r* = 0.331, *p* < 0.001, *r* = 0.500, *p* < 0.001, respectively), it was not significantly correlated with serum magnesium in the present patients with type 2 diabetes. Additional studies are needed to investigate the relationship among mineral associated parameters, including calcium, magnesium, and zinc, in various atherothrombotic stages evaluated by PWV or IMT.

## 5. Conclusions

In summary, serum zinc was found as an independent factor associated positively with serum T_50_ in patient with type 2 diabetes mellitus. Furthermore, zinc was shown to decrease the propensity of calcification in serum in healthy volunteers as well as patients undergoing hemodialysis

## Figures and Tables

**Table 1:** Clinical characteristics of the study participants (*n* = 132).

Measurement	Median (IQR) or Percentage
Age (years)	71 (65–75)
Male/female, *n* (%)	78 (59.1)/54 (40.9)
Body mass index (kg/m^2^)	24.5 (21.8–27.0)
T_50_ (min)	306 (269–332)
eGFR (mL/min/1.73 m^2^)	59.0 (37.6–73.9)
Creatinine (mg/dL)	0.87 (0.69–1.36)
Blood urea nitrogen (mg/dL)	17 (15–23)
Serum albumin (g/dL)	4.1 (3.8–4.3)
Fasting plasma glucose (mg/dL)	119 (99–143)
HbA1c (%)	8.0 (7.1–9.2)
Corrected calcium (mg/dL)	9.4 (9.2–9.7)
Phosphate (mg/dL)	3.8 (3.4–4.2)
Magnesium (mg/dL)	2.1 (2.0–2.3)
Zinc (µg/dL)	80.0 (70.1–89.8)
Whole PTH (pg/mL)	21.6 (16.4–31.2)
Intact PTH (pg/mL)	36 (27–54)
Urine albumin to creatinine ratio (mg/gCr)	17 (6–212)
Systolic blood pressure (mmHg)	125 (110–138)
Diastolic blood pressure (mmHg)	65 (60–74)
Current smoker (%)	65 (49.2)
Use of mediations	
Antihypertensive (%)	79 (59.8)
Statin (%)	64 (48.4)
Insulin (%)	63 (47.7)
Anti-diabetic agent (%)	98 (74.2)
Complications	
Retinopathy (%)	37 (28.2)
Neuropathy (%)	66 (50.0)
Any prior cardiovascular disease (%)	21 (15.9)

The table gives number and percentage for categorical variables and median (IQR) for continuous variables. Abbreviations are: IQR, interquartile range; eGFR, estimated glomerular filtration rate; HbA1c, hemoglobin A1c; PTH, para-thyroid hormone.

**Table 2:** Correlation of serum T_50_ with clinical factors in diabetic patients.

Clinical Variables	Correlation with T_50_	
	*r_s_*	*p*
Age	−0.222	0.010
Body mass index	0.013	0.886
eGFR	0.199	0.022
Creatinine	−0.170	0.051
Blood urea nitrogen	−0.213	0.011
Serum albumin	0.313	0.0003
Fasting plasma glucose	0.282	0.001
HbA1	0.166	0.057
Corrected calcium	0.132	0.132
Phosphate	−0.227	0.009
Magnesium	0.113	0.195
Zinc	0.219	0.012
Whole-PTH	−0.117	0.183
Intact-PTH	−0.110	0.183
Urine albumin to creatinine ratio	−0.247	0.004

Data include the Spearman’s correlation coefficient (*r_s_*-value) and the levels of significance (*p*-value) (bolded if *p* < 0.05). Abbreviations are: eGFR, estimated glomerular filtration rate; HbA1c, hemoglobin A1c; PTH, para-thyroid hormone.

**Table 3:** Factors associated with serum calcification propensity (T_50_) in 132 type 2 diabetes patients.

	Model 1		Model 2	
	*β*	*p*	*β*	*p*
Age	−0.077	0.412	−0.056	0.544
Sex (female = 0. male = 1)	0.042	0.633	0.035	0.692
Any Prior cardiovascular disease	−0.143	0.112	−0.139	0.117
Current smoking (no = 0, yes = 1)	−0.142	0.094	−0.135	0.105
eGFR	0.172	0.155	0.096	0.438
Urinary albumin-creatinine ratio	0.004	0.968	0.028	0.783
Corrected calcium	0.228	0.010	0.199	0.021
Phosphate	−0.328	0.0007	−0.317	0.0008
Magnesium	0.226	0.009	0.216	0.011
Zinc	0.213	0.038	0.229	0.024
HbA1c	–0.054	0.537	-	-
Fasting plasma glucose	-	-	0.171	0.053
R^2^	0.244		0.265	
	(*p* < 0.001)		(*p* < 0.001)	

Data are the standard regression coefficients (*β*-value) and levels of significance (*p*-value) (bolded if *p* < 0.05). Abbreviations are: HbA1c, hemoglobin A1c; R2, multiple coefficient of determination; -, fasting plasma glucose and HbA1c were not included as explanatory variables in the mode 1 and 2, respectively.

## Supplementary Materials

The following are available online at https://www.mdpi.com/2227-9059/8/9/337/s1, Figure S1: Schematic illustration of zinc and calcification; Table S1: Clinical characteristics of the pooled serum from healthy volunteers and patients undergoing hemodialysis. References [38,51] are cited in the supplementary materials.
