# Supplementary material for: Association between Serum Zinc and Calcification Propensity (T50) in Patients with Type 2 Diabetes Mellitus and In Vitro Effect of Exogenous Zinc on T50
Source: Biomedicines. 2020 Sep 9;8(9):337. doi: 10.3390/biomedicines8090337 (PMC7555216; doi:10.3390/biomedicines8090337)
Supplement: Supplementary file 1 [file biomedicines-08-00337-s001.pdf]

biomedicines

Supplementary Materials

Association Between Serum Zinc and Calcification Propensity (T_{50}) in Patients With Type 2 Diabetes Mellitus and In Vitro Effect of Exogenous Zinc on T_{50}

Shinya Nakatani [1], Katsuhito Mori [2,*], Mika Sonoda [1,3], Kozo Nishide [1], Hideki Uedono [1], Akihiro Tsuda [1], Masanori Emoto [1,2] and Tetsuo Shoji [4,5]

[1] Department of Metabolism, Endocrinology and Molecular Medicine, Osaka City University Graduate School of Medicine, Osaka, 1-4-3 Asahi-machi, Abeno-ku, Osaka 545-8585, Japan; m2026719@med.osaka-cu.ac.jp (S.N.); mksnd1110@gmail.com (M.S.); westoutdedede@yahoo.co.jp (K.N.); uedono1217@yahoo.co.jp (H.U.); naranotsudadesu@infoseek.jp (A.T.); memoto@med.osaka-cu.ac.jp (M.E.)
[2] Department of Nephrology, Osaka City University Graduate School of Medicine, Osaka, 1-4-3 Asahi-machi, Abeno-ku, Osaka 545-8585, Japan
[3] Division of Internal Medicine, Inoue Hospital, 16-17 enoki-machi, 564-0053 Suita, Osaka, Japan
[4] Department of Vascular Medicine, Osaka City University Graduate School of Medicine, Osaka, Japan, 1-4-3 Asahi-machi, Abeno-ku, Osaka 545-8585, Japan; t-shoji@med.osaka-cu.ac.jp
[5] Vascular Science Center for Translational Research, Osaka City University Graduate School of Medicine, Osaka, 1-4-3 Asahi-machi, Abeno-ku, Osaka 545-8585, Japan
* Corresponding author: ktmori@med.osaka-cu.ac.jp; Tel.: +81-6-6645-3806; Fax: +81-6-6645-3808

Supplemental Table S1. Clinical characteristics of the pooled serum from healthy volunteers and patients with hemodialysis.

Measurement	Pooled Sample from Healthy Volunteers	Pooled Sample from Hemodialysis Patients
Creatinine (mg/dL)	0.83	9.92
Serum albumin (g/dL)	4.7	3.5
Corrected calcium (mg/dL)	9.6	9.4
Phosphate (mg/dL)	3.2	5.7
Magnesium (mg/dL)	2.4	2.6
Zinc (μg/dL)	105	66

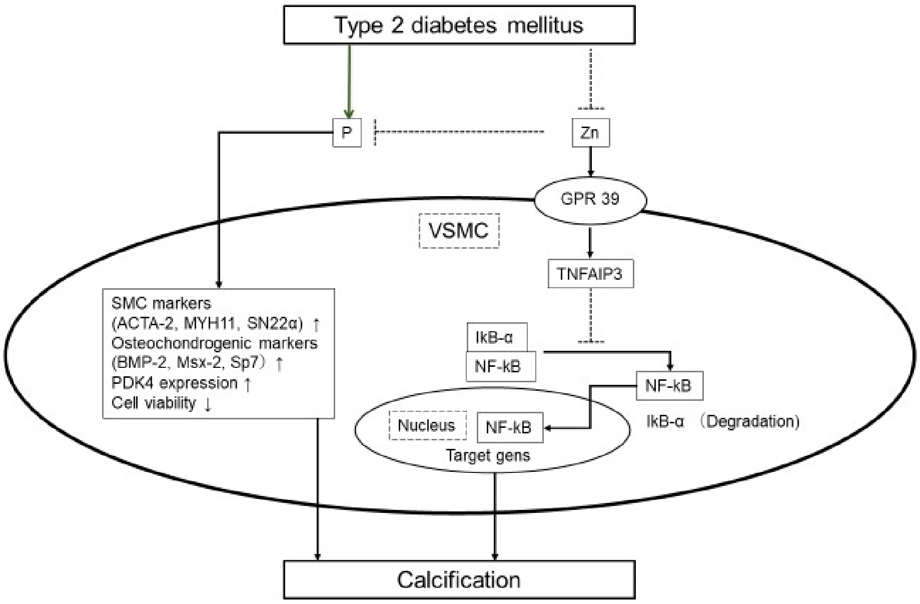

Supplemental Figure S1. Schematic illustration of zinc and calcification.

Type 2 diabetes mellitus induces hypozincemia and hyperphosphatemia. In vascular smooth muscle cells (VSMCs), exposure to elevated phosphate levels induces NF-kB activation. That active NF-kB transcription factor translocates to the nucleus and induces target gene expression to promote osteo/chondrogenic trans-differentiation of VSMCs, leading to calcification. Zinc supplementation may increase zinc finger protein TNF-α-induced protein 3 (TNFAIP3) levels by upregulating zinc-sensing receptor ZnR/GPR39-dependent TNFAIP3 gene expression. Increased TNFAIP3 inhibits NF-kB activation and osteo-/chondrogenic reprograming, resulting in suppression of phosphate-induced VSMC calcification [38].

Zinc also inhibits osteochondrogenic phenotypic switch of VSMCs, reflected by a lower phosphate uptake, thus decreasing the osteochondrogenic gene expressions of Msx-2, BMP-2, and Sp7, as well inducing loss of smooth muscle cell-specific markers. Zinc preserves the phosphorylation state of Runx2 and Ser451, decreases pyruvate dehydrogenase kinase 4 (PDK4) level, and restores cell viability [51].
